# Regulation and outcomes of localized RNA translation

**DOI:** 10.1002/wrna.1721

**Published:** 2022-02-14

**Authors:** Alexander N. Gasparski, Devon E. Mason, Konstadinos Moissoglu, Stavroula Mili

**Affiliations:** ^1^ Laboratory of Cellular and Molecular Biology, Center for Cancer Research National Cancer Institute, NIH Bethesda Maryland USA

**Keywords:** cytoskeleton, local translation, mechanical signaling, RNA localization, RNA transport

## Abstract

Spatial segregation of mRNAs in the cytoplasm of cells is a well‐known biological phenomenon that is widely observed in diverse species spanning different kingdoms of life. In mammalian cells, localization of mRNAs has been documented and studied quite extensively in highly polarized cells, most notably in neurons, where localized mRNAs function to direct protein production at sites that are quite distant from the soma. Recent studies have strikingly revealed that a large proportion of the cellular transcriptome exhibits polarized distributions even in cells that lack an obvious need for long‐range transport, such as fibroblasts or epithelial cells. This review focuses on emerging concepts regarding the functional outcomes of mRNA targeting in the cytoplasm of such cells. We also discuss regulatory mechanisms controlling these events, with an emphasis on the role of cell mechanics and the organization of the cytoskeleton.

This article is categorized under:

Translation > Regulation
RNA Export and Localization > RNA Localization

## Graphical abstract

The cellular transcriptome adopts a variety of localization patterns. mRNA translation at particular cytoplasmic locations is important to mediate functional outcomes and can be controlled by mechanical signaling and the organization of the cytoskeleton.

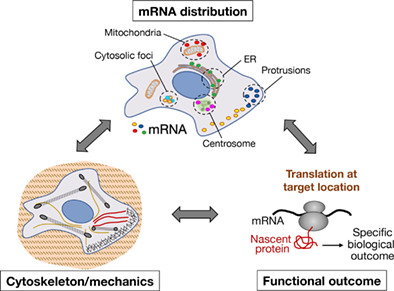

## 1 INTRODUCTION

mRNA molecules in the cytosol of eukaryotic cells often do not adopt a diffuse distribution but are concentrated in specific subcellular locations. Such localized mRNAs have been studied extensively in model organisms, where they have essential roles in development and cell fate determination. Classic examples are found in *Drosophila* and *Xenopus* oocytes and early embryos (such as the *oskar*, *nanos*, *bicoid*, and *Vg1* mRNAs), as well as in budding yeast (such as the *Ash1* mRNA; Buxbaum et al., 2015; Medioni et al., 2012; Paquin & Chartrand, 2008). These systems are characterized by their large size and/or stable polarization. For example, a *Drosophila* oocyte is about 500 μm in length and 200 μm wide, while a mature *Xenopus* oocyte can have a diameter of more than 1 mm. Here, mRNAs are thought to be transported in a silent state and are translationally activated at a specific location or developmental stage, thus allowing for precise spatial and temporal control of protein synthesis (Besse & Ephrussi, 2008). A similar picture has been described for contributions of localized mRNAs in the physiology of mammalian neuronal cells. Numerous mRNAs are actively targeted to axons or dendrites of neurons and have important roles during axonal pathfinding and synaptic plasticity. In neuronal cells, where axons and dendrites can be projected up to meters away from the soma, transporting and maintaining mRNAs in a silent state and activating them upon specific stimulation (Buxbaum et al., 2014; Colak et al., 2013; Koppers et al., 2019) can again allow protein synthesis to occur at distal locations in a controlled manner (Holt et al., 2019; Holt & Schuman, 2013; Rangaraju et al., 2017). Global studies have provided some support for this view (Glock et al., 2021; Perez et al., 2021; Zappulo et al., 2017). This mode of regulation can provide significant advantages. Transporting an mRNA which can locally produce multiple protein molecules can be energetically favorable over transport of individual proteins, and it allows for rapid responses by quickly changing the local proteome upon receipt of signals at distal locations.

By contrast, in smaller, non‐neuronal cell types, such as mesenchymal and epithelial cells, the usefulness of active mRNA targeting is less apparent. These cells are typically only a few tens of microns in diameter, thus post‐translational protein sorting mechanisms and simple diffusion could be sufficient to guide proteins to their sites of action. Additionally, polarization of such cells can be very dynamic. For example, migrating cells polarize into a leading front and a trailing tail, but as a cell explores its environment, a cytoplasmic region can convert from an extending leading front to a retracting tail within minutes. Therefore, the functional advantage of actively targeting mRNAs to inherently transient cytosolic regions has been unclear. Nevertheless, several studies in recent years have demonstrated that a large fraction of mRNAs exhibit specific distribution patterns in the cytosol of mesenchymal and epithelial cells. As detailed below, these distributions can be associated with specific organelles, cytosolic foci, or functionally defined cytoplasmic regions such as protrusions of migrating cells or apical and basal compartments of epithelia.

This review details evidence indicating that mRNAs in non‐neuronal cells primarily move in the cytoplasm in a translationally active state, in contrast to the model supported by paradigms studied in large and stably polarized systems. In fact, translation is required for many mRNAs to reach their destination and co‐translational interactions of the nascent chain can play an active role in the process. For example, organelle‐associated mRNA localization is, at least partially, a secondary by‐product of coupling between protein targeting mechanisms and co‐translational events to ensure protein sorting at the right location. Interestingly, in other cases, translation is observed but is not required for trafficking. Furthermore, there are instances where mRNA distribution does not coincide or explain the distribution of its encoded protein, hinting toward additional roles for mRNA localization. In this regard, recent work suggests that mRNA localization can serve to direct local co‐translational interactions. These interactions can support the efficient assembly of multiprotein complexes, or, in the case of proteins that can have multiple interacting partners, they can guide the selection of interactors and thus influence the functional potential of the locally encoded polypeptide. The latter type of mechanism has the potential to be utilized for the modulation of protein activity. We discuss examples where, indeed, the transport and/or local translation of such mRNAs is regulated based on the properties of the extracellular environment and the mechanical state of the cell.

## 2 RNA LOCALIZATION AT ORGANELLES OR DEFINED CYTOPLASMIC STRUCTURES

### 2.1 Endoplasmic reticulum

**FIGURE 1:**
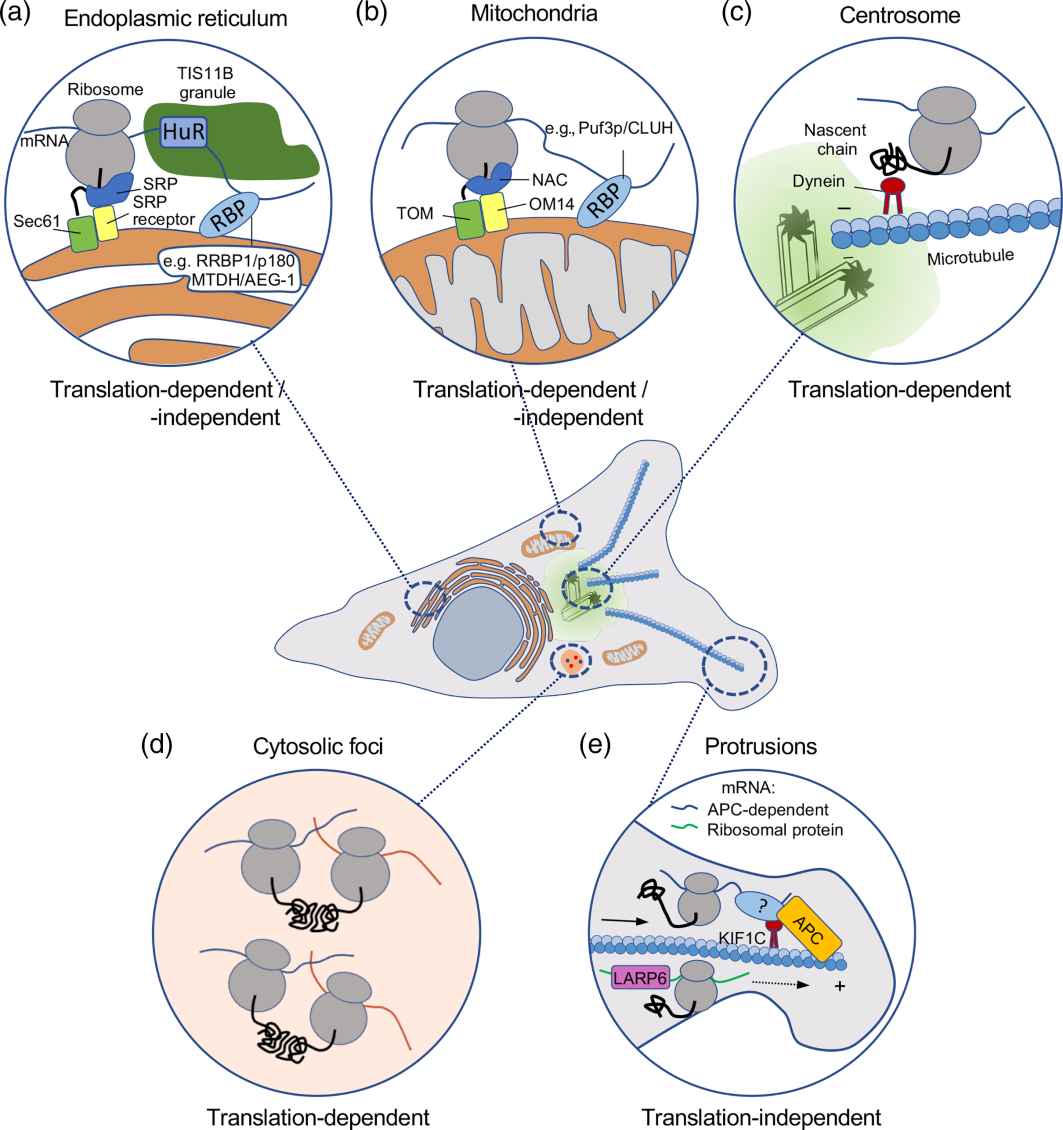
mRNAs accumulate in various cytoplasmic regions and localization patterns display distinct dependencies on protein synthesis. Schematic depicting cellular regions where mRNAs can localize. Insets detail associations with membrane‐bound organelles, such as the endoplasmic reticulum (a) and mitochondria (b); defined membrane‐less structures, such as centrosomes (c); or cytosolic regions, such as foci (d) or cell protrusions (e), which are not associated with a specific organelle or structure. While in all cases mRNAs are undergoing translation (indicated by the appearance of the encoded nascent polypeptide [solid black line]), targeting can be translation‐dependent or translation‐independent, as indicated. Protein factors involved in each localization mechanism are shown and discussed in the text

Several studies have revealed the existence of a large population of endoplasmic reticulum (ER)‐associated mRNAs (Chartron et al., 2016; Diehn et al., 2006; Fazal et al., 2019; Jan et al., 2014; Kaewsapsak et al., 2017). Several of these mRNAs encode secreted or transmembrane proteins, however soluble cytosolic proteins can also be synthesized on the ER (Hoffman et al., 2019; Jan et al., 2014; Lerner et al., 2003; Reid & Nicchitta, 2015; Voigt et al., 2017). Multiple mechanisms can lead to mRNA targeting on the ER surface. According to the classic pathway, mRNAs for secreted or transmembrane proteins are targeted to the ER through recognition of a signal sequence or transmembrane domain within the nascent polypeptide by the signal recognition particle (SRP). This interaction stalls translation elongation allowing the SRP–ribosome–nascent chain–mRNA complex to be recruited to the ER surface by the SRP receptor. The nascent polypeptide is subsequently channeled toward the ER lumen through the Sec61 translocon (Walter & Johnson, 1994; Figure 1a). Translation‐ and nascent chain‐dependent mRNA localization to the ER can also occur without a signal peptide. For example, the *DIAPH1/Dia1* mRNA is targeted to ER membranes through interaction of the nascent N‐terminal GTPase‐binding domain with perinuclear active RhoA (Liao et al., 2011). In the above cases, mRNAs are thought to be passively targeted to the ER as a consequence of their translation.

Nevertheless, mRNAs can additionally have a more active role in ER localization (Kraut‐Cohen & Gerst, 2010; Weis et al., 2013). Such RNA‐based ER targeting can underlie the ER association of mRNAs encoding soluble cytosolic proteins (Pyhtila et al., 2008), but can additionally further support the targeting of mRNAs for secreted proteins. Indeed, pyrimidine‐rich motifs present either within the untranslated or the coding regions of mRNAs promote ER association and enhance the secretion of encoded proteins (Cohen‐Zontag et al., 2019). Additionally, signals within the 3′UTR of mRNAs can support the recruitment of the SRP to ribosomes even prior to synthesis of the signal sequence and can thus direct membrane localization of the mRNA (Chartron et al., 2016).

Such cis‐acting RNA regulatory sequences are likely recognized by ER‐localized proteins with RNA‐binding activity (Bethune et al., 2019). These include the RRBP1/p180 protein and MTDH/AEG‐1, which preferentially associates with ER resident mRNAs (Cui et al., 2012; Hsu et al., 2018). Numerous additional ER integral membrane proteins display RNA binding activity, suggesting the existence of multiple mechanisms for mRNA anchoring on the ER surface (Jagannathan, Hsu, et al., 2014). In a distinct mechanism, mRNAs for membrane associated proteins which also contain AU‐rich elements within their 3′UTRs, are recognized by the TIS11B RNA‐binding protein. TIS11B and RNA–RNA interactions drive the partitioning of mRNAs into the TIGER domain, a mesh‐like RNA condensate that intertwines along the ER surface (Ma et al., 2021; Ma & Mayr, 2018; Figure 1a).

Localization to the ER can functionally affect an mRNA and its encoded protein in multiple ways. Certainly, the localization of secreted and transmembrane protein mRNAs on the ER facilitates co‐translational translocation and correct protein maturation. Interestingly, however, SRP targeting can additionally prevent mistargeting to mitochondrial membranes (Costa et al., 2018; see also below). Furthermore, single‐molecule imaging of translation of an ER‐associated mRNA encoding a cytosolic protein has detected a difference in the translation rate of ER‐associated compared with cytosolic mRNA molecules (Voigt et al., 2017) indicating that the ER environment can alter the translational output of specific transcripts. Finally, ER‐associated mRNAs are underrepresented in stress‐induced granules (Khong et al., 2017; Unsworth et al., 2010), suggesting a regulatory role of ER association upon stress.

### 2.2 Mitochondria

The majority of mitochondrial proteins are encoded by nuclear mRNAs and need to be imported into the organelle. Mitochondrial proteins encoded by nuclear genes carry targeting signals and can be imported post‐translationally. Nevertheless, several of their mRNAs are associated with the mitochondrial outer membrane in yeast and animal cells and are translated on its surface (Fazal et al., 2019; Gadir et al., 2011; Kaewsapsak et al., 2017; Williams et al., 2014). Similar to ER‐associated mRNAs, accumulation of mRNAs on mitochondria seems to be a result of both co‐translational targeting through recognition of the nascent chain as well as targeting through sequences in the mRNA itself (Figure 1b). The translocase of the outer membrane (TOM) complex, which interacts with ribosomes (Gold et al., 2017) and the chaperone protein Ssa1, which recognizes the amphipathic helix of the mitochondrial targeting signal peptide, are both involved in targeting translating ribosomes to the mitochondrial surface (Eliyahu et al., 2010; Eliyahu et al., 2012). The outer membrane protein OM14 also functions as a receptor for the nascent polypeptide associated complex (NAC) and supports the recruitment of cytosolic ribosomes (Lesnik et al., 2014).

Apart from such co‐translational interactions, which occur after the appearance of the nascent polypeptide, signals within the mRNA sequence can additionally direct targeting to mitochondria (Das et al., 2021; Lashkevich & Dmitriev, 2021; Weis et al., 2013). Such signals are recognized by RNA‐binding proteins on the surface of mitochondria, which often have additional functions in regulating stability or translation of their mRNA targets (Bethune et al., 2019). For example, Puf3p in yeast, a member of the Pumilio homology domain family, associates with the 3′UTR of mRNAs encoding mitochondrial proteins and is required for their correct targeting to the outer mitochondrial surface (Lashkevich & Dmitriev, 2021; Quenault et al., 2011). Puf3p additionally controls stability and translation of its associated RNAs, a function fulfilled by the CLUH protein in higher eukaryotes (Schatton et al., 2017). Several other RNA‐binding proteins on the outer mitochondrial surface can serve as anchors or regulators of translation (Qin et al., 2021; Schatton & Rugarli, 2018).

Localized synthesis of mitochondrial mRNAs on the outer mitochondrial membrane impacts the efficiency of mitochondrial import and coordinates mitochondrial function with growth conditions and recovery from stress. Additionally, RNA targeting and co‐translational import can ensure protein targeting to the correct organelle. As mentioned above, removal of the SRP from cells leads to erroneous targeting of ER proteins to mitochondria, causing mitochondrial fragmentation (Costa et al., 2018). Similarly, the nascent polypeptide associated complex (NAC) has been implicated in the recruitment of translating ribosomes to the mitochondrial membrane (George et al., 1998; George et al., 2002) and in suppressing nonspecific interactions of nascent polypeptides with the SRP receptor (Hsieh et al., 2020). Depletion of the NAC causes erroneous localization of ribosomes synthesizing mitochondrial proteins to the ER membrane and incorrect import to the ER lumen leading to a strong impairment in protein homeostasis in both organelles and a reduction in life span (Gamerdinger et al., 2015). In an analogous way, inactivation of the COPI vesicle coat complex decreases localization of mRNAs encoding mitochondrial proteins to mitochondria, while instead increasing their localization to the ER. This results in deficits in membrane potential and respiratory growth defects (Zabezhinsky et al., 2016). While the mechanistic details are unclear, the picture that emerges is that, for membrane‐bound organelles, an important role for RNA targeting is to ensure that the encoded proteins are imported into the right organelle.

RNA targeting and local translation at an organelle‐specific level can also be potentially utilized to promote repair of individual mitochondria. The kinase PINK1 might participate in such a mechanism. PINK1 binds to mRNAs encoding respiratory chain complex subunits, and in cooperation with Tom20 localizes them to the outer mitochondrial membrane, where they are translationally activated through displacement of repressors including Pumilio and Glorund/hnRNP‐F (Gehrke et al., 2015). This translational upregulation has been proposed to be utilized to promote repair of mildly damaged mitochondria (Wu et al., 2018). On the other hand, upon persistent damage, PINK1 leads to ribosome stalling, recruitment of factors of the no‐go decay pathway and clearance by mitophagy (Wu et al., 2018). Therefore, mRNA targeting and controlled translation on the surface of the organelle can allow for responses that are tailored to the functional status of individual mitochondria (Kejiou & Palazzo, 2017).

### 2.3 Centrosomes

The centrosome serves as the primary microtubule‐organizing center in most animal cells and is composed of a central pair of centrioles surrounded by a proteinaceous matrix of pericentriolar material (PCM). Several mRNAs have been shown to be associated with centrosomes in diverse cell types including early embryos and cultured mammalian cells (Chouaib et al., 2020; Lecuyer et al., 2007). Many, but not all, centrosomal mRNAs encode centrosome components or regulators of centrosome function. Interestingly, mRNA localization to centrosomes is regulated during the cell cycle. Localization of specific mRNAs can vary between interphase and mitosis, or can be altered at different mitotic stages, seemingly correlating with cell‐cycle dependent changes in PCM composition and functional changes in centrosome activity (Ryder et al., 2020; Safieddine et al., 2021).

A general emerging picture is that mRNA delivery to centrosomes requires translation and involves the nascent polypeptide. This is based on the fact that centrosomal localization of several mRNAs requires intact polysomes since it is disrupted even by a brief treatment with puromycin or harringtonine, compounds which lead to dissociation of ribosomal subunits, but not by treatment with cycloheximide or emetine, which block translation elongation without dissociating the ribosomal subunits and releasing the nascent chains (Safieddine et al., 2021; Sepulveda et al., 2018). Further supporting the dependence on translation, the coding sequence of centrosome localized mRNAs is necessary and sufficient to direct targeting to centrosomes and its translation is important since introduction of an early termination codon suppresses this effect (Safieddine et al., 2021). The nascent protein likely mediates transport of polysomes using cytoplasmic dynein or through tethering to microtubules, which are pulled toward the centrosome (Safieddine et al., 2021; Sepulveda et al., 2018; Figure 1c). It is thought that such co‐translational delivery of nascent proteins to centrosomes facilitates their correct incorporation and centrosome maturation, akin to the role suggested for co‐translational interactions for the correct assembly of multi‐subunit complexes (Kramer et al., 2019; Schwarz & Beck, 2019; Williams & Dichtl, 2018; see also next section). Indeed, localization of *centrocortin* (*cen*) mRNA in Drosophila embryos is necessary for Cen protein localization to centrosomes and disruption of this mechanism leads to errors in spindle assembly and mitotic defects (Ryder et al., 2020). Not all centrosomal components, however, are encoded by localized mRNAs. What determines the necessity for mRNA localization is unclear, however potentially pertinent to this point, most of the studied centrosomally targeted mRNAs encode for proteins of relatively large sizes (Safieddine et al., 2021; Sepulveda et al., 2018). It is possible that coupling their incorporation into the centrosome with local synthesis overcomes the challenges associated with correct folding of large proteins.

While translation appears to be commonly required for mRNA targeting to centrosomes, further mechanistic details appear to be different for specific mRNAs. For example, components of the exon junction complex are required for *NIN* mRNA localization at centrosomes at the base of cilia but are dispensable for targeting of *BICD2* mRNA at the same site (Kwon et al., 2021). Furthermore, apart from translation‐dependent localization, mRNAs can be targeted to centrosomes in additional ways. In an interesting example, Bergalet et al. showed that *cen* mRNA secondarily directs localization of its cis‐natural antisense mRNA *ik2*, encoding the IκB kinase like‐2 protein. The coding region of *cen* mRNA is sufficient for its own targeting. However, an additional region in the 3′UTR is complementary to a corresponding region included in the 3′UTR of its natural antisense *ik2* mRNA. This shared region mediates a physical interaction between the two mRNAs and is required for centrosomal targeting of *ik2* mRNA either through co‐transport or association with already targeted *cen* mRNAs (Bergalet et al., 2020).

Co‐translational complex assembly is likely not the only role fulfilled by centrosomal mRNAs. Indeed, not all mRNAs localized at centrosomes encode proteins that associate with the organelle, suggesting that centrosomal mRNA targeting might mediate additional effects. In a mollusk embryo, mRNAs for patterning factors, including orthologues of the BMP2 and BMP4 (bone morphogenetic protein) family of secreted ligands, and of the BMP1 metalloprotease, which can modulate TGFβ signaling, are associated with centrosomes during early embryonic cleavage cycles. In this case, centrosomal association leads to asymmetric segregation of these mRNAs between daughter cells, potentially contributing to embryonic patterning (Lambert & Nagy, 2002).

## 3 RNA LOCALIZATION TO SPECIFIC CYTOSOLIC REGIONS

### 3.1 RNA foci/assemblysomes

Several recent studies have highlighted that groups of mRNAs can undergo compartmentalized translation in the cytosol in the absence of an apparent association with a defined cytoplasmic structure or organelle. In these cases, mRNAs organize in nonmembrane bound foci or assemblysomes. Localized translation in these structures is linked with the productive assembly of a multisubunit complex or with the metabolism of the nascent peptide rather than with directly determining the localization of the mature protein.

For example, transcription factors and co‐activators, such as TFIID, TREX‐2, and SAGA, are large complexes composed of multiple subunits. Translating mRNAs for specific subunits of these complexes physically associate and can co‐localize in the cytosol. The resulting co‐translational interaction of nascent partner proteins is important to avoid nonspecific interactions or protein aggregation (Kamenova et al., 2019; Kassem et al., 2017). In an analogous way, the Rpt1 and Rpt2 subunits of the proteasome, in yeast and human cells, are encoded by mRNAs that colocalize in distinct cytoplasmic particles. These are distinct from other types of cytosolic granules, such as stress granules or P‐bodies, and contain the Not1 protein, the scaffold of the Ccr4–Not complex, and are dependent on it for their formation. Co‐translational interaction of the nascent Rpt1 and Rpt2 proteins within these Not‐1 containing assemblysomes promotes their productive association and likely ensures accurate assembly of the proteasome (Panasenko et al., 2019). In these cases, mRNA segregation seems to be primarily driven by the nascent protein without any indication of active mRNA transport (Figure 1d). The underlying mechanism rather presents parallels to the classic protein sorting pathway to the ER. Indeed, translation pauses after exposure of the N‐terminal interacting domain in nascent Rpt1. Interaction with its Rpt2 partner results in resumption of translation likely within the assemblysome (Panasenko et al., 2019). Thus, in both cases translational pausing is used to ensure that the nascent peptide has engaged in interaction with the appropriate partner, or at the appropriate location, prior to resumption of translation.

A global screen has recently revealed that several mRNAs, such as *BUB1*, *DYNC1H1*, and *CTNNB1*, accumulate in distinct foci that do not colocalize with each other or with other known granules such as P bodies. Formation of these mRNA foci is dependent on translation and the mRNAs are translated within foci, indicating that mRNA foci correspond to specialized translation factories (Chouaib et al., 2020). The underlying mechanism leading to mRNA accumulation in foci is unclear, but in the case of the *CTNNB1* mRNA it could potentially involve liquid–liquid phase separation. *CTNNB1* encodes the transcription factor β‐catenin, whose degradation or nuclear translocation is controlled by the destruction complex, a multiprotein assembly that targets β‐catenin for phosphorylation and eventual degradation. Components of the destruction complex include the scaffold protein Axin, the tumor suppressor APC, and the kinases GSK3β and CK1α. Axin and APC are large proteins that contain multiple protein binding domains and intrinsically disordered regions which drive condensation and phase separation of the destruction complex in the cytosol (Nong et al., 2021; Schaefer & Peifer, 2019). Interestingly, core components of the destruction complex accumulate in *CTNNB1* mRNA foci, indicating that these foci likely correspond to destruction complex condensates and reflect sites where β‐catenin is co‐translationally degraded (Chouaib et al., 2020). Consistent with this, *CTNNB1* mRNA foci disperse upon Wnt signaling activation concomitant with stabilization of β‐catenin (Chouaib et al., 2020).

Recruitment of *CTNNB1* mRNA in these cytoplasmic foci is likely not absolutely required for β‐catenin degradation, since destruction complex phase separation and β‐catenin phosphorylation can occur in vitro in the absence of *CTNNB1* mRNA or active translation (Nong et al., 2021). Nevertheless, it is conceivable that recruitment of the *CTNNB1* mRNA and co‐translational association of the nascent protein with the destruction complex in vivo provides tighter control and minimizes leakage of β‐catenin into the nucleus in the absence of Wnt signaling (Chouaib et al., 2020). The efficiency of such a mechanism would presumably rely on the relative kinetics of *CTNNB1* mRNA translation versus its recruitment in destruction complex foci. Given that the phosphorylation sites relevant for degradation are found at the N‐terminus of β‐catenin, it would be interesting to determine whether translational pausing after emergence of the N‐terminus might contribute to tighter control, similar to the cases described above. It is also still unclear whether additional mRNAs encoding other potential destruction complex substrates (Kim et al., 2009) are included in these foci, or if there is specificity for *CTNNB1* mRNA recruitment, and how this is determined, against other RNAs that can associate with the APC protein (Mili et al., 2008; Preitner et al., 2014) and might be predicted to phase separate in the same structures.

### 3.2 Protrusion‐localized mRNAs

mRNAs can additionally segregate in cytosolic regions which are defined functionally rather than through the presence of a specific structure. An example is RNA localization at protrusive cytosolic regions. Such protrusions are prominently observed in migrating cells and include a wide range of diverse peripheral cytosolic regions, such as broad lamellipodia that are generated at the front of moving cells, narrow filopodia and other extensions formed at the leading front or retracting tail of cells. Protruding cellular regions can be mechanically isolated using microporous filters (Cho & Klemke, 2002). This methodology has been used in several studies to identify mRNAs enriched in cell protrusions (reviewed in Herbert & Costa, 2019). These studies have revealed that a subset of cellular mRNAs become enriched in protrusions through distinct pathways, which can depend on active transport on cytoskeletal elements.

One prominent pathway is defined by its dependency on the APC tumor suppressor protein and targets to protrusions mRNAs such as *RAB13*, *NET1*, *KIF1C*, *TRAK2*, and several others, with roles in membrane trafficking, cytoskeletal regulation, and transport (Mili et al., 2008; Wang et al., 2017). This group of mRNAs has been termed APC‐dependent and their localization is prominently observed at protrusive regions of numerous normal or cancerous cell types (Chouaib et al., 2020; Costa et al., 2020; Mardakheh et al., 2015; Moissoglu et al., 2020; Wang et al., 2017) as well as at the invasive front of in vivo tumors (Chrisafis et al., 2020). While the role of the APC protein is still not well defined, APC physically associates with these mRNAs (Mili et al., 2008), likely through its ability for direct RNA binding (Baumann et al., 2020; Preitner et al., 2014). APC‐dependent mRNAs share other features that underlie their coregulation. They are targeted to protrusions in an active manner that requires the kinesin KIF1C motor and a specific subset of stable microtubules that are modified by detyrosination (Pichon et al., 2021; Wang et al., 2017; Yasuda et al., 2017). RNAs are trafficked on microtubules in association with KIF1C and, similar to what has been described for all the other cases above, actively translating, polysome‐associated RNAs can serve as the cargo (Moissoglu et al., 2019; Figure 1e). Nevertheless, active translation or the identity of the coding region is not required for trafficking. Consistent with that, the 3′UTRs of mRNAs in this group are sufficient to direct localization to protrusions and GA‐rich regions play an important role (Costa et al., 2020; Mili et al., 2008; Moissoglu et al., 2020; Pichon et al., 2021).

mRNAs can be targeted to protrusions in additional ways, independent of APC. Interestingly, while APC‐dependent mRNAs correspond to relatively low‐abundance transcripts, other protrusion transcripts studied are among the most abundant cellular mRNAs. A widely studied example is the β‐actin mRNA, which localizes to lamellipodial regions of migrating cells using a zipcode sequence in its 3′UTR and a combination of microtubule‐ and/or actin‐dependent transport depending on the cell type (Condeelis & Singer, 2005; Nalavadi et al., 2012; Song et al., 2015). Another RNA group targeted at protrusions of virtually all migrating cells includes mRNAs encoding for ribosomal proteins, the protein constituents of ribosomal subunits (Dermit et al., 2020; Mardakheh et al., 2015; Moriarty et al., 2021; Wang et al., 2017). Protrusion localization of ribosomal protein mRNAs is mediated through the TOP sequence at their 5′UTR and relies on the LARP6 protein (Dermit et al., 2020; Figure 1e).

**FIGURE 2:**
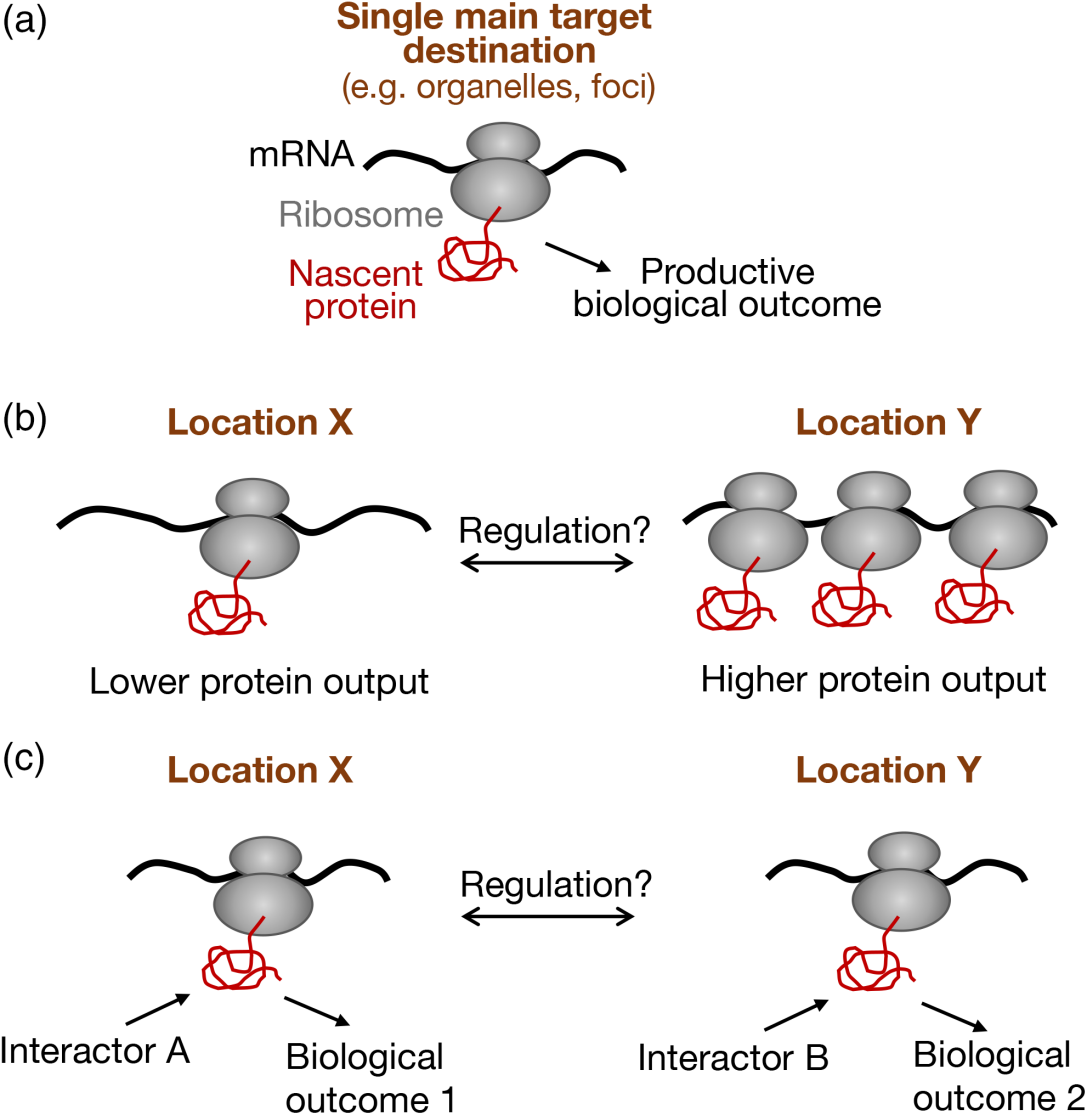
Biological outcomes upon translation of localized mRNAs. (a) For mRNAs with a single main target destination, translation at the target site can act as a fail‐safe mechanism that ensures a productive biological outcome. For example, mRNA targeting to the endoplasmic reticulum, or on the surface of mitochondria, ensures proper maturation of the encoded polypeptide and its incorporation into the correct organelle. In an analogous way, concentration of mRNAs encoding partner subunits into cytoplasmic foci allows co‐translational interactions that promote correct assembly of multiprotein complexes. Absence of such a mechanism has deleterious consequences. (b) The presence of an mRNA at different cytosolic locations, X and Y, can affect the efficiency of its translation and the corresponding protein output. For example, the β‐actin and ribosomal protein mRNAs exhibit increased translation when found at protrusive lamellipodial regions of migrating cells. Altering the fraction of mRNA distributed between sites X and Y could be used to regulate cognate protein levels. (c) Translation of an mRNA at different cytosolic locations, X and Y, can tune the functional potential of the encoded polypeptide. For example, the *RAB13* mRNA, which encodes a protein that can engage with multiple interacting partners (either activators or effectors), can be translated in either peripheral or perinuclear regions. Translation at a particular cytosolic location introduces the newly synthesized polypeptide into a local network of potential interactors, favoring certain interactions over others. Altering the fraction of mRNA distributed between sites X and Y can thereby shift the balance between biological outcomes

mRNA targeting to cell protrusions is important for cell movement. For example, delocalization of β‐actin mRNA from the leading edge affects cell motility (Condeelis & Singer, 2005; Song et al., 2015). Similarly, disrupting the localization of APC‐dependent mRNAs as a group, or perturbing the localization of individual transcripts, causes defects in cell migration, cancer cell invasion and blood vessel morphogenesis (Chrisafis et al., 2020; Costa et al., 2020; Moissoglu et al., 2020; Wang et al., 2017). However, the specific molecular consequences that bring about these phenotypic effects can vary. Furthermore, in this regard, protrusion‐localized mRNAs present mechanistic paradigms that are distinct from those presented in the previous sections. In the cases described previously, there appears to be a functionally “correct” localization, which can support a productive biological outcome, while in its absence there are deleterious consequences (Figure 2a). For instance, inability to target mRNAs to the ER can compromise protein maturation and trafficking; mistargeting of mitochondrial or centrosomal mRNAs can negatively affect the function of the respective organelles; inability of large protein complexes to co‐translationally assemble in cytoplasmic foci can lead to protein aggregation and degradation. By contrast, protrusion‐localized RNAs can produce functional proteins in both internal and peripheral cytosolic locations. Interestingly, the exact site of translation rather serves to either influence the metabolism of the mRNA itself (Figure 2b), or to modulate the activity of the nascent protein and thus the balance between biological outcomes (Figure 2c).

An example of the first scenario is provided by the abundant, APC‐independent localized transcripts. Here, cytosolic location appears to affect mRNA translational output. Specifically, translation of β‐actin mRNA is induced in lamellipodial regions by Src‐induced phosphorylation and dissociation of the translational repressor ZBP1 (Huttelmaier et al., 2005). Similarly, localization of ribosomal protein mRNAs at protrusions correlates with increased translation, through unknown mechanisms, leading to enhanced ribosome biogenesis and overall upregulation of protein synthesis (Dermit et al., 2020). Therefore, in these cases transport of an mRNA to a peripheral cytosolic location affects the metabolism of the mRNA itself (Figure 2b).

In the case of APC‐dependent mRNAs, RNA localization appears to serve a different role. Transport of APC‐dependent mRNAs is not linked to changes in their translational output (Moissoglu et al., 2019) but rather alters the functional potential of the nascent polypeptide (Moissoglu et al., 2020; Figure 2c). The molecular mechanism has been investigated in some detail for the case of the APC‐dependent mRNA encoding the RAB13 GTPase, a member of the Rab family of GTPases which controls the trafficking of various molecules that affect cell migration, organization, and proliferation (Ioannou & McPherson, 2016). RAB13, as other Rab proteins, cycles between an active, GTP‐bound state and an inactive, GDP‐bound state, a conversion catalyzed through the action of guanine nucleotide exchange factors (GEFs) and GTPase activating proteins (GAPs), respectively. Rab proteins can be regulated by various GEFs and GAPs and can signal downstream through several effectors (Muller & Goody, 2018; Pylypenko et al., 2018). Therefore, the temporal and spatial regulation of Rab activation and their association with specific cellular membranes is central to their ability to mediate specific functional effects. For RAB13 this regulation is at least partly achieved through localization of its mRNA. Specifically, peripheral translation of the *RAB13* mRNA allows a co‐translational interaction of nascent RAB13 with one of its activators, RABIF, and the location of this interaction is required to direct RAB13 activity toward migration relevant effectors (Moissoglu et al., 2020). Disruption of *RAB13* mRNA localization phenocopies an almost complete RAB13 protein loss when assaying for cell migration (Moissoglu et al., 2020), underscoring the importance of mRNA localization in controlling RAB13 function in this context. While perinuclearly‐translated RAB13 cannot support cell migration, it is likely still functional since it remains substantially active (i.e., GTP‐loaded) and associates normally with several of its regulatory partners. These studies thus suggest that altering the site of RAB13 protein synthesis can serve as a regulatory mechanism that favors interaction of the nascent protein with specific regulators and effectors, thus promoting specific functional outcomes. Consistent with this idea, perinuclear‐ or peripherally‐translated RAB13 engages with different interacting networks (Moissoglu K, unpublished data). Such a mechanism could provide a broadly applicable selection method for proteins that have the potential to engage with multiple interacting partners (Figure 2c).

In a potentially analogous mechanism, the mRNA encoding the transcription factor C/EBPβ can be synthesized either peripherally or perinuclearly, controlled by the RNA‐binding protein HuR and oncogene expression. Perinuclear translation allows phosphorylation of C/EBPβ by ERK1/2 and alters C/EBPβ specificity toward its transcriptional targets (Basu et al., 2011). Pointing toward further generality of this type of regulation, global studies have shown that for the majority of protrusion‐localized mRNAs there is little correlation between mRNA enrichment and steady‐state concentration of the corresponding proteins (Mardakheh et al., 2015). Therefore, it is likely that in contrast to the foci or assemblysomes mentioned above, in these instances the exact site of mRNA translation in the cytosol is important, not for locally increasing the cognate protein concentration or for promoting the formation of a single functional complex, but rather to provide a way of tuning the balance among multiple potential functional interactions and outcomes (Figure 2c). It might be interesting to speculate that, in a similar manner, cytosolic proteins which can be translated either by free or ER‐bound ribosomes (Jagannathan, Reid, et al., 2014) might be directed in each case to distinct functional paths.

## 4 MECHANICAL REGULATION OF RNA TRAFFICKING AND TRANSLATION

In several instances mRNAs reach specific cytoplasmic destinations and are locally translated as a result of active transport on cytoskeletal elements. mRNAs can serve as direct or indirect cargo of molecular motors, such as kinesins, dynein, and myosin, which promote movement along microtubules or actin filaments. mRNAs engaged in such transport events usually contain specific sequence elements or zipcodes that direct their association with molecular motors. Current knowledge regarding RNA transport complexes has been previously reviewed (Bullock, 2011; Gagnon & Mowry, 2011). An important consideration for understanding mRNA transport is that the cytoskeletal elements used for molecular transport are not stable linear tracks but dynamic structures that adapt to the extracellular environment. For example, microtubules can be dynamically unstable, undergoing rounds of growth and depolymerization, or they can exist in stable forms that are marked by various post‐translational modifications (Janke, 2014; Roll‐Mecak, 2020). Actin filaments can form a highly branched network that supports broad lamellipodial regions or can organize in bundles that can withstand large tensile forces (Svitkina, 2018). This structural diversification, apart from providing a range of tracks for molecular transport, allows the cytoskeleton to additionally confer mechanical stability to the cell and is controlled by the mechanical state of the environment through intricate mechanosensitive signaling pathways. Interestingly, RNA metabolism and translation as well as remodeling of the cytoskeleton are among the most energy‐demanding cellular processes that utilize a large fraction of available ATP (Bernstein & Bamburg, 2003; Buttgereit & Brand, 1995; DeWane et al., 2021). It is thus maybe not surprising that mechanisms exist to coordinately regulate these processes. Indeed, increasing evidence suggests that mechanical stimuli can control both the cytoskeleton as well as the transport and translation of localized mRNAs.

### 4.1 Mechanical control of RNA transport

Cells sense external forces and the mechanical properties of their environment and respond by initiating a series of mechanotransduction events and modulating their contractility (Hoffman et al., 2011). A central component of such signaling pathways is the RhoA GTPase, whose effectors, such as the kinase ROCK and the formin mDia1 subsequently affect the properties of both the actin and microtubule cytoskeleton (Bartolini & Gundersen, 2010; Chesarone et al., 2010; Lessey et al., 2012). Early studies pointed toward a connection between mechanical forces and RNA localization by showing that application of mechanical tension to cell surface integrin receptors promotes the recruitment of ribosomes and polyadenylated RNAs at the site of stress (Chicurel et al., 1998). The connections between mechanical properties and RNA localization have been studied predominantly for protrusion‐localized mRNAs in the context of cell migration, a process that relies on and is centrally controlled by physical forces (Gardel et al., 2010).

**FIGURE 3:**
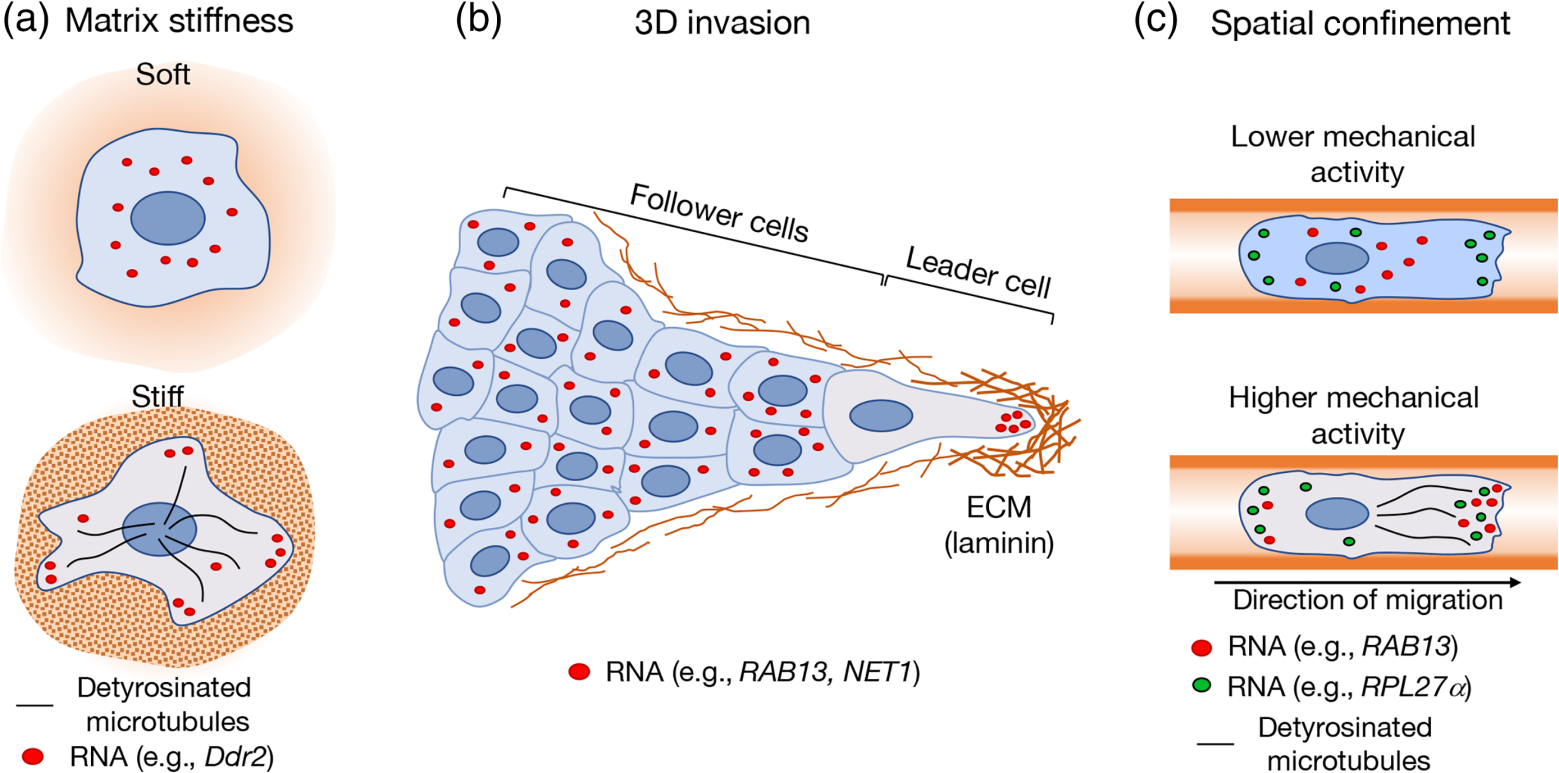
Mechanical regulation of mRNA localization. (a) The stiffness of the extracellular matrix modulates the distribution of cytosolic mRNAs. Stiff environments promote protrusion localization of APC‐dependent mRNAs (such as *Ddr2*) via a RhoA‐dependent mechanism that involves detyrosination of the microtubule network. (b) Localization patterns differ among cells in collectively invading 3D groups. During collective invasion of cancer cells, APC‐dependent mRNAs (such as *NET1* and *RAB13*) localize prominently at the front of leader cells. This localization requires integrin‐mediated adhesion to the extracellular matrix (ECM) and coincides with a local accumulation of extracellular laminin. Notably, such an mRNA localization pattern is not observed in follower cells. (c) Spatial confinement modulates mRNA localization patterns. Ribosomal protein mRNAs (such as *RPL27α*) become more enriched at peripheral protrusions of cells migrating in confining microchannels. The localization of other mRNAs in confinement depends on the mechanical state of the cell. APC‐dependent mRNAs (such as *RAB13*) become preferentially enriched at leading protrusions of cell types that exhibit higher mechanical activity and stable, detyrosinated microtubules. This localization pattern can contribute to the efficiency of cell migration through confined spaces

Activation of the RhoA GTPase and myosin function were shown to be required for localization of the β‐actin and Arp2/3 subunit mRNAs in lamellipodia. Similarly, RhoA activity, through ROCK and myosin II activation, promoted the microtubule independent accumulation of mRNAs in protrusions of tumor cells (Latham et al., 2001; Mingle et al., 2005; Stuart et al., 2008). Furthermore, APC‐dependent mRNAs have been preferentially found in protrusions enriched for Ser19‐phosphorylated myosin light chain, a modification associated with higher contractile activity. Indeed, RhoA activity and actomyosin contractility are required for the localization of APC‐dependent mRNAs to cell protrusions (Wang et al., 2017). In this case, the role of RhoA is likely dual as another RhoA effector mDia1 functions to promote formation of stable, detyrosinated microtubules which are required for transport of APC‐dependent mRNAs to peripheral protrusions. This mechanism can be regulated by and in response to the stiffness of the extracellular substrate (Wang et al., 2017; Figure 3a).

Cell migration in confined or three‐dimensional (3D) environments provides additional cues that influence cytoskeletal dynamics and direct the mode of movement adopted by cells (Friedl & Alexander, 2011; Paul et al., 2017). These cues influence mRNA transport and determine the contribution of localized mRNAs to different modes of migration. In a 3D model of collective cancer cell invasion, the APC‐dependent mRNAs *RAB13* and *NET1* accumulate at the front of leader cells in invasive cell strands. This accumulation is important for efficient invasion. RNA accumulation at the invasive front requires integrin‐mediated contact with the extracellular matrix and coincides with areas of high laminin concentration (Chrisafis et al., 2020). Interestingly, such polarized mRNA distribution is not observed in follower cells (Chrisafis et al., 2020), suggesting that adhesion to a denser matrix and the higher traction forces exerted by leader cells (Lintz et al., 2017; Reffay et al., 2014) underlie this preferential mRNA localization pattern (Figure 3b). In addition, cancer cells exposed to the mechanical cue of spatial confinement exhibit different mRNA localization patterns, likely determined by the mechanical state of the cell (Moriarty et al., 2021). Cells with mechanosensitive ion channels and increased myosin II activity maintain a detectable network of detyrosinated microtubules and support the peripheral localization of the *RAB13* APC‐dependent mRNA. This localization is in turn functionally important for the ability of cells to move efficiently in confinement. By contrast, cells that exhibit lower mechanical activity do not support peripheral *RAB13* mRNA localization, due to low levels of detyrosinated microtubules (Figure 3c). These cells move through confined channels using a migration mode that does not depend on *RAB13* mRNA localization (Moriarty et al., 2021). Overall, mechanical stimuli encountered by migrating cells alter the cytoskeleton to promote mRNA transport toward peripheral destinations and support efficient cell movement.

Increased contractility and mechanical activity do not indiscriminately promote mRNA transport to protrusions, but rather appear to increase trafficking of specific mRNAs such as those belonging to the APC‐dependent group. In contrast, mRNAs encoding ribosomal proteins preferentially accumulate in less contractile protrusions (Wang et al., 2017). Interestingly, the localization of ribosomal protein mRNAs responds to confining forces. In confinement, ribosomal protein mRNAs become prominently peripheral, however in this case the mechanical state of the cell does not influence this phenotype (Moriarty et al., 2021). While this differential regulation of ribosomal protein mRNA transport is intriguing, the underlying mechanistic basis and functional significance during cell migration are still unclear.

### 4.2 Cytoskeletal control of RNA translation

**FIGURE 4:**
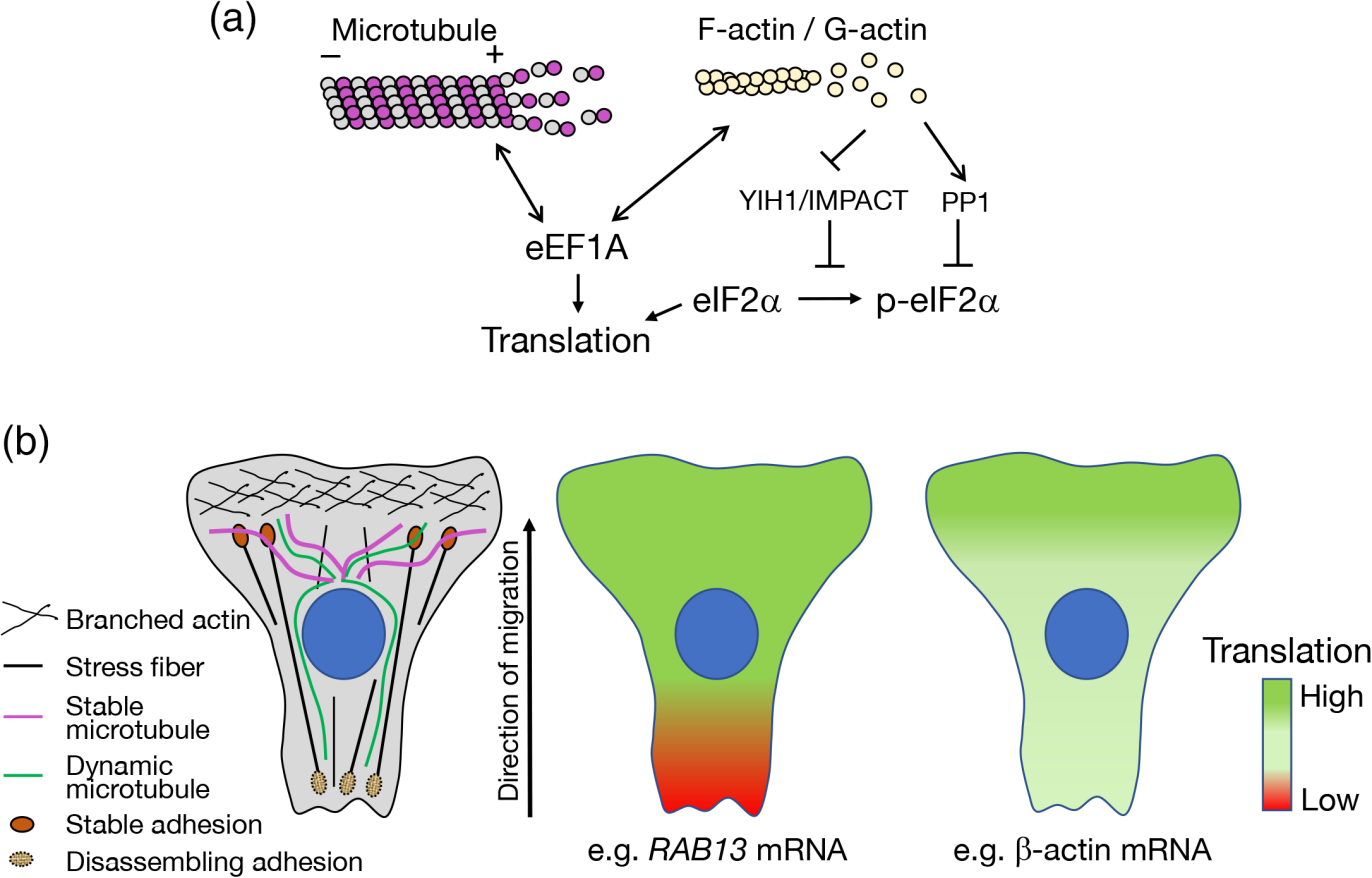
Crosstalk between mRNA translation and the cytoskeleton. (a) Potential connections between the cytoskeleton and translation factors are shown. The translation elongation factor eEF1A can bind and regulate both microtubules and actin filaments. eEF1A binding to the cytoskeleton can likely reciprocally influence eEF1A's function in translation elongation. Phosphorylation of the translation initiation factor eIF2α is controlled by proteins (such as YIH1/IMPACT and PP1) whose activity is regulated by binding to monomeric G‐actin and can thus be influenced by the polymerization state of the actin cytoskeleton (see text for details). (b) Schematics depicting potential connections between local cytoskeletal organization and cytosolic domains associated with low or high translation of specific mRNAs. Left panel: Polarized migrating cells exhibit heterogeneous organization of the cytoskeleton with branched actin enriched in protrusive lamellipodia, stable microtubules oriented toward the front, and actin stress fibers attached to stable adhesions at the front or disassembling adhesions at the back. Right panels: Heat maps representing efficiency of mRNA translation along a migrating cell. The *RAB13* mRNA is translated with similar efficiency in perinuclear regions and peripheral extending protrusions, while it is silenced at retracting areas. β‐actin mRNA translation is upregulated in the vicinity of focal adhesions at protrusive lamellipodia

Apart from serving to transport and anchor mRNAs, the cytoskeleton can spatially organize key components of the translation machinery. Early fractionation studies revealed that 25%–40% of polysomes are bound to the cytoskeleton. Translation initiation and elongation factors, as well as aminoacyl‐tRNA synthetases are also physically linked with cytoskeletal elements (reviewed in Kim & Coulombe, 2010). The resulting regulation appears to be reciprocal. On one hand, translation factors can directly affect the cytoskeleton. A quite well studied example involves the translation elongation factor eEF1A which has a canonical role in binding and delivering cognate aminoacylated‐tRNAs to the A site of the ribosome to advance translation elongation. eEF1A has however, multiple additional connections with the cytoskeleton. eEF1A interacts with microtubules and acts to either stabilize or sever them depending on the context (Sasikumar et al., 2012). eEF1A is additionally an actin binding protein that can both bind and bundle actin filaments in vitro (Sasikumar et al., 2012). Such multifunctionality offers opportunities for coordinating translation and cytoskeletal organization in a global or local manner, however how this is mechanistically achieved is unclear (Figure 4a). Interestingly, binding of eEF1A to actin and microtubules is mediated through the same protein domains, while binding of eEF1A to F‐actin and aminoacylated‐tRNA are mutually exclusive and regulated by pH (Liu et al., 1996). eEF1A is additionally the target of multiple post‐translational modifications (Hamey & Wilkins, 2018) suggesting the potential existence of multiple points for controlling and coordinating the translation and cytoskeletal functions of eEF1A (Mateyak & Kinzy, 2010).

On the other hand, the state and structural integrity of the cytoskeleton impacts on overall translation and supports localized translational responses (Kim & Coulombe, 2010; Piper et al., 2015). Increasing evidence suggests that the cytoskeleton participates in controlling RNA translation through regulation of the activity of the translation initiation factor eIF2α (Figure 4a). Phosphorylation of eIF2α is a key regulatory mechanism for adjusting protein synthesis in response to internal or external cues (Sonenberg & Hinnebusch, 2009). eIF2a phosphorylation is carried out by one of four members of the eIF2α kinase family leading to a decrease in global protein synthesis and initiation of the integrated stress response (Jackson et al., 2010). One of the eIF2α kinases, GCN2, is activated by amino acid deprivation but can additionally be controlled by the state of the actin cytoskeleton. GCN2 activity requires binding to its coactivator GCN1. YIH1 in yeast, and its mammalian homologue IMPACT, acts as a negative regulator of the GCN2 kinase by preventing formation of the GCN1–GCN2 complex (Roffe et al., 2013; Sattlegger et al., 2004). Interestingly, YIH1 binding to monomeric G‐actin prevents its inhibitory action on GCN2 (Sattlegger et al., 2011). Indeed, modulation of actin dynamics alters protein complexes that control GCN2 activity and promotes an eIF2 response (Silva et al., 2016). Given that the dynamics of actin polymerization can be altered in a localized manner, it is possible that a spatially restricted release or sequestration of YIH1/IMPACT could lead to localized fluctuations in protein synthesis in coordination with the state of the actin cytoskeleton in the vicinity. In yeast, it has been suggested that this mechanism might be relevant to increase translation in the vicinity of the growing bud. Cortical actin patches near the growing bud, where filamentous actin is concentrated, could serve as sites where YIH1 is displaced from actin leading to translational upregulation (Sattlegger et al., 2011). Additional ways of integrating actin dynamics with eIF2α regulation center on the function of the phosphatase that reverses eIF2α phosphorylation. This role is fulfilled by the catalytic subunit of the PP1 phosphatase in complex with the PPP1R15 regulatory subunit. In this case, G‐actin stabilizes and provides substrate specificity to the holocomplex (Chambers et al., 2015; R. Chen, Rato, et al., 2015). The potential for G‐actin to both suppress and increase eIF2α phosphorylation, through PP1 or YIH1/IMPACT respectively, raises the possibility that additional spatial or temporal parameters might further fine tune the eventual impact of the actin cytoskeleton on RNA translation.

The existence of mechanisms connecting the structural state of the cytoskeleton to translational control, together with the highly heterogeneous organization of the cytoskeleton in polarized cells, suggests the potential existence of cytosolic domains with different capacity for protein synthesis (Figure 4b). These domains could dynamically change as cytoskeletal architecture changes in response to external stimuli or intrinsic polarity cues. A potential manifestation of such dynamic local regulation is exhibited by the APC‐dependent *RAB13* mRNA, which is localized at peripheral protrusions of migrating cells. While *RAB13* mRNA is found throughout the cell periphery, its translation is spatially regulated, coincident with front‐back polarity (Moissoglu et al., 2019). Specifically, *RAB13* mRNA is translated in extending protrusive areas, while it is translationally repressed in retracting regions. Interestingly, this translational silencing additionally coincides with segregation in multimeric mRNA clusters, reminiscent of phase‐separated RNA granules, such as stress granules, that are formed upon global translational inhibition (Moissoglu et al., 2019). Protrusive lamellipodia have also been linked to increased translation of other mRNAs. Src kinase activation, which occurs upon integrin engagement, upregulates translation of the β‐actin mRNA by alleviating repression through ZBP1 (Huttelmaier et al., 2005). Consistently, β‐actin mRNA near focal adhesions exhibits subdiffusive corralled movement characteristic of increased translation (Katz et al., 2016). Furthermore, localization of ribosomal protein mRNAs to the protrusive front enhances their translation (Dermit et al., 2020). Therefore, in a polarized migrating cell, the protrusive front could correspond to a more translationally permissive domain compared with the contractile, retracting back (Figure 4b). An analogous asymmetry in activity of the translational machinery has been observed between apical and basal compartments of intestinal epithelia (Moor et al., 2017). To what extent such domains affect translation in general or are transcript‐specific, as well as the exact molecular mechanisms, remain to be elucidated.

## 5 FUTURE PERSPECTIVES

It is becoming apparent that the cellular transcriptome is highly compartmentalized in the cytoplasm. Apart from active mRNA transport that supports protein synthesis at distal locations, mRNAs also adopt numerous distribution patterns that are required for function of the encoded protein. RNA translation and co‐translational events play important roles either to actively drive an mRNA to its destination, or to influence a protein's interacting partners based on the local environment encountered by the nascent polypeptide. These mechanisms offer several potential points for regulatory control. As an emerging theme, the mechanical environment through ensuing changes of the local cytoskeletal organization can influence mRNA metabolism, either through alteration of the cytoskeletal tracks used for active transport or through crosstalk with translation factors and potential creation of local translational domains.

Several questions remain: what are the types of mRNA movements that support localization at various cytoplasmic regions; how are they kinetically coordinated with other co‐regulated transcripts; how are mRNA distributions modulated in physiological settings and by relevant stimuli; how prevalent and consistent are localization patterns in cells of different types of polarity and in diverse tissue contexts; can they be used to predict and control protein functions? Emerging methodologies that achieve high‐resolution, multiplexed RNA detection will allow a better understanding of regulatory connections and functional implications (K. H. Chen, Boettiger, et al., 2015; Cho et al., 2021; Eng et al., 2019; Lee et al., 2014). They also raise the need for development of analysis methods to detect and analyze complex distribution patterns and connections between them (Samacoits et al., 2018). Sensitive imaging tools that allow detection of translation at a single molecule level can offer unprecedented opportunities for deep understanding (Morisaki & Stasevich, 2018; Pichon et al., 2018) but might also have limitations in recapitulating the in vivo regulation. Continuous progress in the development of methods that allow imaging with reduced intervention would provide valuable alternatives (Braselmann et al., 2020; Yang et al., 2019).

## CONFLICT OF INTEREST

The authors have declared no conflicts of interest for this article.

## AUTHOR CONTRIBUTIONS

**Alexander N Gasparski:** Writing – original draft (equal); writing – review and editing (equal). **Devon E Mason:** Writing – original draft (equal); writing – review and editing (equal). **Konstadinos Moissoglu:** Writing – original draft (equal); writing – review and editing (equal). **Stavroula Mili:** Conceptualization (lead); writing – original draft (lead); writing – review and editing (lead).

## RELATED WIREs ARTICLES

mRNA localization as a rheostat to regulate subcellular gene expression.

Classical and emerging techniques to identify and quantify localized RNAs.

Following the messenger: Recent innovations in live cell single molecule fluorescence imaging.

Localization elements and zip codes in the intracellular transport and localization of messenger RNAs in Saccharomyces cerevisiae

RNA localization in prokaryotes: Where, when, how, and why.

## Data Availability

Data sharing is not applicable to this article as no new data were created or analyzed in this study.
